# Temporal Trends and Geographic Clustering of U.S. Weather-Related Disasters (1989–2019): A Foundational Baseline for Healthcare Preparedness

**DOI:** 10.3390/ijerph23080984

**Published:** 2026-07-29

**Authors:** Roberta Lavin, Su Zhang, Yue Feng, Xi Gong, Yiliang Zhu, Wei Fang, Xiaozhong Yu, Shuguang Leng, Kritim Bastola, Bhawana Kafle, Daylin Clifton, Shawn L. Penman, Sandeep Talasila, Mary Pat Couig, José M. Cerrato

**Affiliations:** 1 Center for Health Equity: Planetary Health and Preparedness, College of Nursing, University of New Mexico, 2300 Tucker Rd., Albuquerque, NM 87131-0001, USA; rlavin@salud.unm.edu (R.L.); wfang@salud.unm.edu (W.F.); xiyu@salud.unm.edu (X.Y.); kbastola@salud.unm.edu (K.B.); bhkafle@salud.unm.edu (B.K.); dsclifton@salud.unm.edu (D.C.); 2 Department of Geography and Environmental Studies, University of New Mexico, MSC01 1110, 1 University of New Mexico, Albuquerque, NM 87131-0001, USA; suzhang@unm.edu; 3 Earth Data Analysis Center, University of New Mexico, MSC01 1110, 1 University of New Mexico, Albuquerque, NM 87131-0001, USA; spenman@edac.unm.edu (S.L.P.); stalas1@unm.edu (S.T.); 4 Department of Civil, Construction, and Environmental Engineering, University of New Mexico, MSC01 1070, 1 University of New Mexico, Albuquerque, NM 87131-0001, USA; jcerrato@unm.edu; 5 Comprehensive Cancer Center, University of New Mexico, 2325 Camino de Salud, Albuquerque, NM 87131, USA; yfeng9500@gmail.com (Y.F.); sleng@salud.unm.edu (S.L.); 6 Department of Biobehavioral Health, The Pennsylvania State University, 219 Biobehavioral Health Building, University Park, PA 16802, USA; xigong@psu.edu; 7 Institute for Computational and Data Sciences (ICDS), The Pennsylvania State University, 571 Eisenhower Rd., Computer Building, University Park, PA 16802, USA; 8 Department of Internal Medicine, School of Medicine, University of New Mexico, MSC08 4720, 1 University of New Mexico, Albuquerque, NM 87131-0001, USA; yizhu@salud.unm.edu

**Keywords:** Federal Emergency Management Agency, disaster declarations, extreme weather, climate change, healthcare preparedness, nursing workforce, public health

## Abstract

**Highlights:**

**Public health relevance—How does this work relate to a public health issue?**

- The rising frequency and geographic spread of FEMA-declared disasters reflects a growing climate-driven public health crisis that directly impacts population health, healthcare system stability, and community resilience.
- Nurses and public health practitioners are central to disaster preparedness and response; understanding longitudinal disaster trends is essential for informing workforce education and training, and protecting populations disproportionately affected by climate-related events.

**Public health significance—Why is this work of significance to public health?**

- This study provides a comprehensive, longitudinal analysis of U.S. disaster trends, characterizing hazard patterns with implications for healthcare system strain and socially vulnerable populations.
- By connecting disaster epidemiology with workforce preparedness and health equity, this work advances the integration of public health, climate science, and nursing education needed to address emerging population health threats.

**Public health implications—What are the key implications or messages for practitioners, policymakers and/or researchers in public health?**

- Nursing and public health education programs should incorporate climate-informed disaster preparedness, including data interpretation, risk communication, and trauma-responsive care, to better prepare the workforce for increasingly frequent disasters.
- Policymakers and health system leaders should use disaster trend data to guide equitable resource allocation, strengthen infrastructure, and prioritize interventions for socially vulnerable populations at greatest risk.

**Abstract:**

Over the past three decades, the United States has experienced a notable increase in weather-related disasters, including hurricanes, floods, tornadoes, wildfires, and severe storms, posing growing challenges to healthcare preparedness and public health systems. This study analyzes Federal Emergency Management Agency (FEMA) disaster declarations from 1989 to 2019 to characterize temporal and geographic trends in weather-related events. Data after 2019 were excluded to avoid confounding effects associated with the COVID-19 pandemic, which disrupted disaster declarations, resource allocation, and healthcare system demands. Using descriptive statistics, generalized linear mixed models, and spatial clustering techniques, we identified substantial increases and nonlinear patterns in disaster declarations, with variation across hazard types and regions. These trends reflect evolving hazard exposure, regional differences, and policy-driven declaration practices. Although this study does not directly measure health outcomes or social vulnerability, the observed patterns have important implications for healthcare system capacity, workforce preparedness, and populations known to be disproportionately affected by disasters. The findings highlight the need for climate-informed training, data-driven preparedness planning, and integration of disaster trend analysis into nursing education and public health practice. Strengthening the ability of healthcare systems to anticipate and respond to evolving disaster patterns is critical for advancing resilience and promoting equitable health outcomes in the context of climate change.

## 1. Introduction

As extreme weather events continue to increase in both frequency and severity, the resulting challenges to health system preparedness, health equity, and community resilience have become increasingly apparent. The National Oceanographic and Atmospheric Administration’s (NOAA) National Centers for Environmental Information (NCEI) has documented the escalating incidence of billion-dollar weather and climate disasters across the United States, highlighting the substantial economic losses associated with these events [1]. Beyond economic impacts, these disasters pose profound threats to population health and the functionality of the healthcare system. Exposure to extreme weather events is associated with elevated risks of cardiovascular, respiratory, and mental health conditions, which in turn lead to increased healthcare utilization and further strain on an already overburdened U.S. healthcare system [2,3]. Older adults, children, individuals with chronic disease, and socially marginalized communities are at increased risk for heat-related illness, exacerbations of cardiovascular and respiratory disease, and disruptions in access to essential healthcare services following disasters [2,3]. These trends underscore the urgent need for comprehensive, evidence-based strategies to protect population health, enhance adaptive capacity, and strengthen the resilience of health systems. These trends underscore the need for health systems that are increasingly resilient, adaptive, and capable of protecting populations disproportionately affected by climate-related disasters. Without proactive planning and targeted investment, the cumulative burden of extreme weather events is likely to escalate, amplifying health disparities and challenging the sustainability of healthcare delivery in the United States [2]. These challenges also have direct implications for nursing education, as nurses must be prepared to respond to increasingly complex, climate-driven public health emergencies across diverse care settings [4].

Nurses comprise the largest segment of the U.S. healthcare workforce and are central to disaster mitigation, preparedness, response, and recovery [4]. National nursing organizations, accreditation standards, and professional codes of ethics emphasize disaster preparedness as a core professional competency [5,6,7].

However, evidence suggests persistent gaps in disaster education and preparedness training, particularly for climate-related hazards that increasingly affect healthcare delivery [8].. Identifying local and regional hazard patterns is therefore essential for preparing the nursing workforce and strengthening healthcare system resilience.

Despite nursing education [5], a professional code of ethics [7] related to disaster preparedness and duty to respond, and healthcare facility accreditation requirements for preparedness [6], there are still gaps in nursing education and workforce training for preparedness. The American Association of Colleges of Nursing, the professional organization that provides standards on baccalaureate and graduate nurse education and competencies, includes competencies for preparedness under the domain of population health [5] (pp. 35–36). These competencies include emergency preparedness, population health assessment, interprofessional collaboration, risk communication and ethical disaster response. The American Nurses Association’s issue brief on nurses and disasters [7] describes nurses’ ethical duty to respond during a disaster and other considerations including legal and moral. The Joint Commission’s National Performance Goal #3 [6] describes the standards for hospitals to meet accreditation requirements. Identifying local and regional risks is an integral part of helping to ensure the healthcare workforce and systems are prepared.

Federal Emergency Management Agency (FEMA) disaster declarations provide one of the most comprehensive national datasets for examining long-term patterns of disasters requiring federal assistance. Under the Robert T. Stafford Disaster Relief and Emergency Assistance Act, federal disaster and emergency declarations are issued when an event exceeds state, territorial, or tribal response capacity and federal support is warranted [9]. Because declarations are recorded using standardized criteria across jurisdictions, FEMA data provide a consistent longitudinal resource for evaluating temporal and geographic trends in weather-related disasters [10]. Characterizing these patterns can help identify regions experiencing changing hazard profiles and provide evidence to support preparedness planning, healthcare system resilience, and workforce education. Despite recognition of the growing disaster burden, prior research has largely examined disaster frequency, hazard trends, or health outcomes in isolation [11,12]. Although prior studies have examined disaster frequency, hazard trends, or health outcomes independently, relatively few studies have characterized long-term national disaster patterns in ways that can serve as a baseline for future analyses of healthcare system preparedness, workforce capacity, social vulnerability and nursing education [13]. This gap limits understanding of how evolving disaster patterns influence healthcare delivery, resource allocation, and population health outcomes, particularly among socially vulnerable populations. Communities with limited economic resources, reduced access to healthcare, higher baseline health risks, or pre-existing infrastructure deficits often face disproportionate health burdens during disaster events. These inequities can exacerbate morbidity, delay recovery, and place additional stress on healthcare systems, creating cycles of vulnerability that are amplified with successive disasters. Moreover, the health impacts of disasters extend beyond physical injury to include mental health challenges, chronic disease exacerbation, and trauma-related outcomes, underscoring the importance of integrating trauma-responsive and climate-informed approaches into healthcare planning and disaster preparedness initiatives.

Recent evidence demonstrates that climate-related disasters substantially disrupt healthcare delivery, workforce capacity, and patient outcomes. A mixed-methods study of Federally Qualified Health Centers (FQHCs) in Puerto Rico and the U.S. Virgin Islands and a systematic review of 46 studies on climate disasters and oncology care report consistent impacts across settings, including facility closures, supply chain disruptions, infrastructure failures, and reduced patient access [14,15]. Workforce preparedness remains limited, with many healthcare workers reporting low confidence and insufficient disaster training. Both studies also highlight the dual burden on clinicians, who must manage personal disaster-related impacts while continuing to provide care. Adverse effects are disproportionately experienced by vulnerable populations, including individuals with chronic conditions and cancer, exacerbating existing health inequities through treatment interruptions and reduced access to services. Despite these insights, the current literature largely focuses on downstream healthcare impacts and does not systematically link temporal and geographic disaster patterns to healthcare system capacity, underscoring the need for integrated, population-level analyses.

To address these gaps, this study characterizes temporal and geo-graphic patterns of FEMA-declared weather-related disasters across the United States from 1989 to 2019, a period reflecting the modern disaster declaration framework established under the Stafford Act. Data after 2019 were excluded because the COVID-19 pandemic substantially altered disaster declarations, resource allocation, and healthcare system operations, creating conditions that could confound long-term trend analyses [16,17]. The objective of this study is to establish a national epidemiologic baseline of weather-related disaster trends and regional hazard patterns that can inform future investigations of healthcare system preparedness, nursing workforce education, and equitable public health planning. By providing a macro-level characterization of disaster patterns, this work offers a foundation for integrating disaster epidemiology with future analyses of healthcare capacity, social vulnerability, and workforce preparedness in the context of a changing climate.

## 2. Materials and Methods

### 2.1. Data Source

Data on federally declared disasters from 1989 through 2019 were obtained from OpenFEMA Data Sets (https://www.fema.gov/about/openfema/data-sets, accessed on 22 May 2026) FEMA, Washington, DC. Data after 2019 are excluded to avoid confounding effects introduced by the COVID-19 pandemic, which caused unprecedented disruptions in disaster declarations, resource allocation, and healthcare system demands [16,17]. The analysis included both Major Disaster Declarations (DRs) and Emergency Declarations (EMs). A Major Disaster Declaration provides a mechanism for comprehensive, long-term federal recovery programs and financial aid for rebuilding infrastructure and assisting individuals after severe damage has occurred. An EM provides immediate, short-term assistance to protect life, property, and public health before or during a crisis [9]. FEMA assigns each declaration a disaster number and declaration type, and declarations were issued for specific states. Therefore, a single physical disaster event affecting multiple states is represented by separate FEMA declaration identifiers for each affected state.

Disaster occurrences were analyzed by calendar year, with each declaration assigned to the year in which it was issued. Events spanning multiple calendar years were counted only once, in the year of declaration. To prepare the data for analysis, several processing criteria were applied. First, only weather-related natural hazard declarations were retained, while human-caused events, including terrorist attacks, hazardous material incidents, and other technological disasters, were excluded. Weather-related disasters included coastal storms, wildfires, floods, freezing events, hurricanes, landslides, severe ice storms, severe convective storms, snowstorms, tornadoes, tsunamis, and droughts. Second, because FEMA issues disaster declarations at the state level, statewide declarations were transformed into county-level records by assigning each declaration to all counties within the affected state. Third, county Federal Information Processing Standards (FIPS) codes (https://www.census.gov/library/reference/code-lists/ansi.html, accessed on 22 May 2026) U.S. Census Bureau and American National Standards Institute (ANSI), Washington, DC, were standardized across the study period to account for historical changes in county identifiers, ensuring that counties with revised FIPS codes were consistently tracked as the same geographic entities and were not mistakenly treated as separate counties. The resulting output was a standardized county-level dataset of weather-related FEMA disaster declarations from 1989 to 2019, with consistent temporal and spatial identifiers, which was used for subsequent spatial and temporal analyses.

### 2.2. Statistical Methods

National trends in annual weather-related disaster occurrences from 1989 to 2019 were first described through visual inspection. Subsequently, temporal trends in disaster occurrences across the 50 US states and the District of Columbia were evaluated using generalized linear mixed-effect models (GLMMs). Since the likelihood of weather-related disasters varies geographically, and disaster management practices differ by state, state-level disaster counts were utilized as the outcome. Time was the main predictor, with random intercepts and random slopes included to account for state-level variation in baseline disaster frequency and temporal trajectories, respectively, and the presence of an excess of zero counts was accommodated. Model specification was guided by assessment of over-dispersion and the linearity of temporal trends. Over-dispersion was evaluated using the Pearson chi-square dispersion statistic, calculated as the sum of squared Pearson residuals divided by the residual degrees of freedom (df). Linearity was explored descriptively through bar plots and formally assessed using likelihood ratio tests (LRTs). To capture potential nonlinear temporal patterns, various GLMMs were compared using: (i) a linear year term, (ii) a basis spline for year (df = 3), that is, a smooth curve that allows the trend to bend slightly over time, and (iii) a combined linear and spline specification. Model fit was evaluated using the Akaike Information Criterion (AIC), the Bayesian Information Criterion (BIC), and LRTs, with *p* < 0.05 indicating significant improvement in model fit. Final model selection was based on convergence behavior, LRT results, and information criteria, with simpler models favored when added complexity did not materially improve fit.

Finally, hierarchical clustering was applied to model-derived estimates to identify groups of states exhibiting similar temporal patterns in disaster occurrence. Clustering was conducted using Euclidean distance, which considers both the overall magnitude and the shape of the temporal trajectories, and Ward’s minimum variance method [18,19], which tends to form compact and homogeneous clusters. The optimal number of clusters was determined by visually inspecting dendrograms and selecting the cut point that balanced between-cluster separation and within-cluster similarity.

All statistical analyses were performed using R 4.5.2 (R Core Team, Vienna, Austria) and the “glmmTMB” (Version 1.1.14) package.

## 3. Results

### 3.1. National Trends in Weather-Related Disaster Declarations

**Figure 1:**
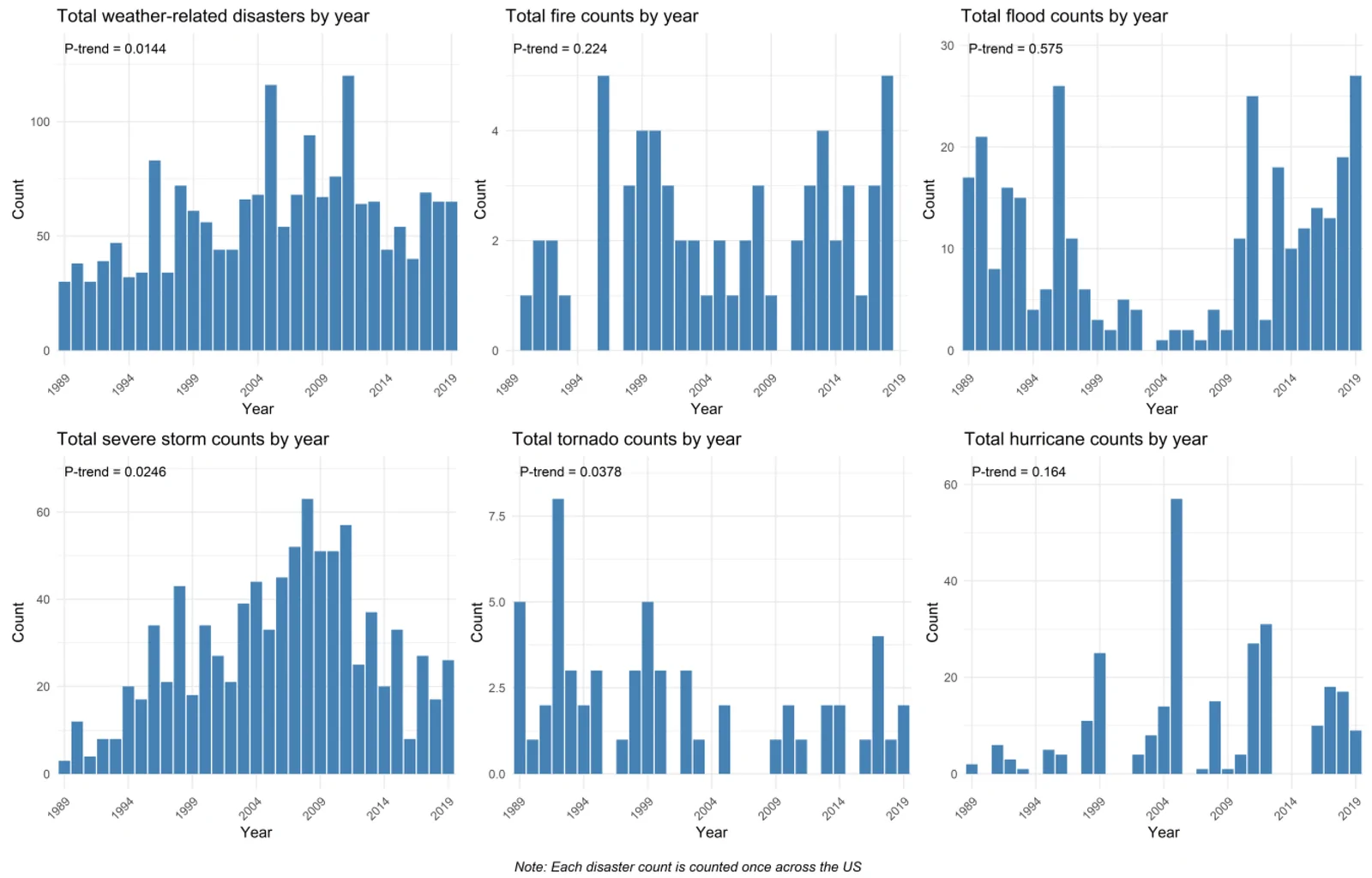
Annual statewide disaster counts by type, from 1989 to 2019. Note: Counts represent one record per disaster per state across the US. The six panels display annual counts for all weather-related disasters combined and for individual categories: fires, floods, severe storms, tornadoes, and hurricanes.

Annual statewide disaster counts by type from 1989 to 2019 are illustrated in Figure 1, where it is evident that the counts fluctuated considerably from 1989 to 2019. In particular, the total number of weather-related disasters increased from 30 in 1989 to 65 in 2019, with notable peaks in 2005 (*n* = 116) and 2011 (*n* = 122). Severe storm occurrences rose from 3 in 1989 to 26 in 2019, peaking between 2007 and 2011. Flood occurrences followed a distinct V-shaped trajectory, with a 10-year low between 1999 and 2009.

Further, Figure S1 (Supplementary Information) illustrates the percent age of US states that experienced weather-related disasters categorized by annual count and type per year. Each disaster is counted once per state, regardless of the number of counties designated within that state.

To reduce short-term annual variability and better visualize broader temporal patterns, the annual statewide disaster counts were further aggregated into five-year intervals, revealing simplified and visually distinct nonlinear trajectories in comparison to the annual counts (Figure S2, Supplementary Information). Data from 1989 were excluded from this analysis to maintain equal interval lengths. In the figure, total weather-related disaster counts peaked in 2005–2009 (*n* = 399), followed by a gradual decline, though remaining higher than levels observed before 2005. Fire counts increased sharply from 1995 to 2004, dipped in 2005–2009, and then gradually rose again in the two most recent periods. Flood counts displayed a pronounced V-shaped pattern, falling from 1990–1994 to 2005–2009, then rebounding markedly in 2010–2014 and 2015–2019. Severe storm counts rose steadily to a maximum in 2000–2004 (*n* = 244) before decreasing in later intervals with the most recent period still exceeding the first. Tornado were most frequent in 1990–1994 (*n* = 16), dropped to a low in 2005–2009 (*n* = 3), and partially recovered thereafter (*n* = 8 in 2015–2019). Hurricane counts fluctuated, with a low in 2000–2004 (*n* = 26) and a peak in 2005–2009 (*n* = 74) before a modest decline.

### 3.2. State-Level Trends in Weather-Related Disaster Occurrences

After evaluating model fit based on over-dispersion parameters (Table S1, Supplementary Information), the high proportion of zero counts (Figure S1, Supplementary Information), linearity, and model convergence (Table S2, Supplementary Information) for each disaster type, specific models were selected. A zero-inflated negative binomial (ZINB) model with a nonlinear spline term was employed for hurricanes; zero-inflated Poisson (ZIP) models with nonlinear spline terms were selected for total weather-related disasters, severe storms, and floods; and ZIP models with linear terms were utilized for fires and tornadoes.

**Figure 2:**
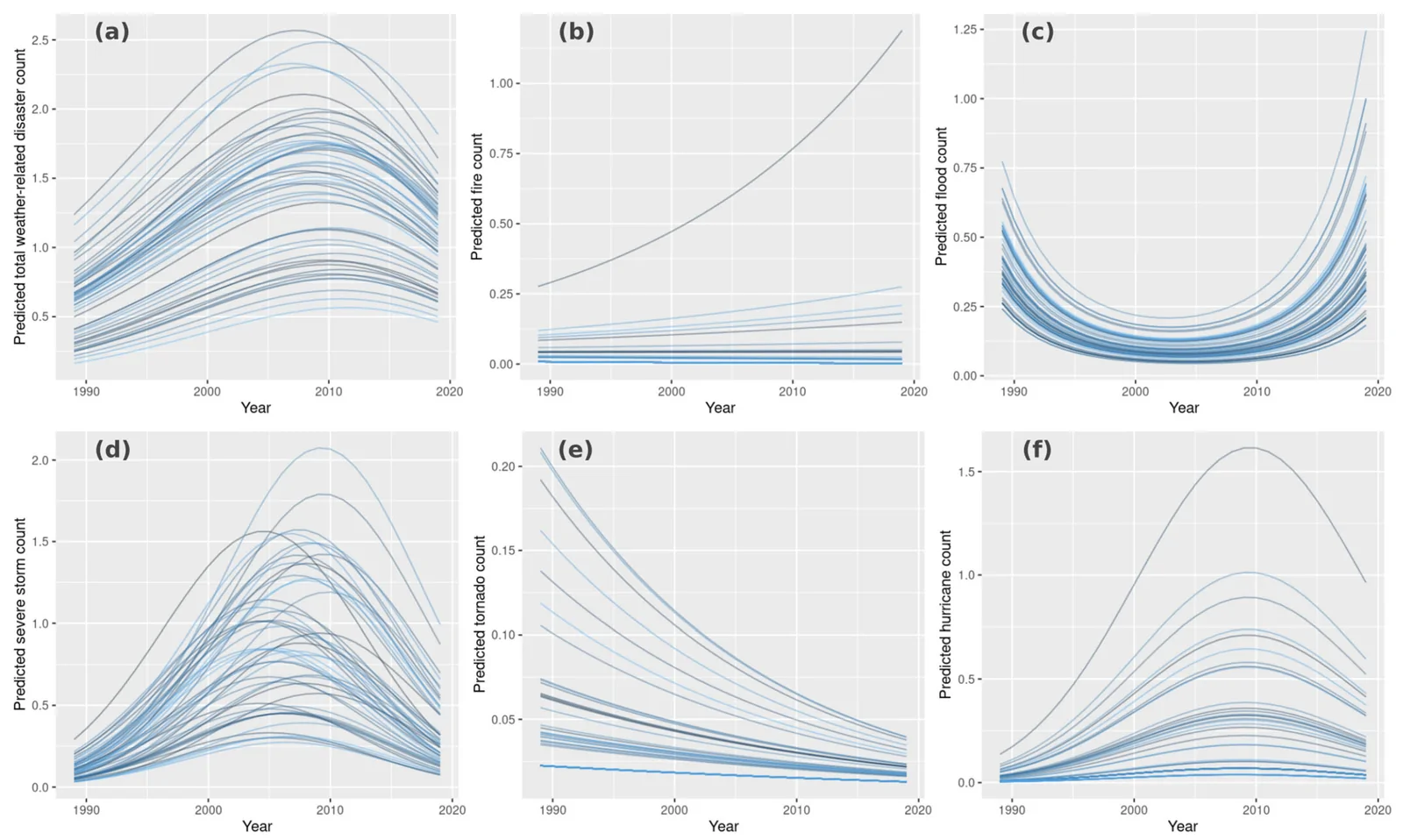
Modeled temporal trends in weather-related disaster occurrences in 50 US states and the District of Columbia, from 1989 to 2019: (**a**) all natural disasters; (**b**) fires; (**c**) floods; (**d**) severe storms; (**e**) tornados; (**f**) hurricanes. Note: Each line represents the model-predicted annual count for an individual state, based on the final selected zero-inflated Poisson (ZIP) or zero-inflated negative binomial (ZINB) mixed-effects models (see Table 1). Predictions account for state-specific random intercepts and slopes, as well as nonlinear temporal effects where applicable.

Based on these model specifications, distinct temporal patterns across disaster types were revealed by the ZIP and ZINB mixed-effects models (Table 1) and the modeled state-specific temporal trajectories were shown in Figure 2.

In particular, in Table 1, while states exhibited significant heterogeneity in baseline disaster frequencies (indicated by substantial random intercept variance), the temporal trajectories were remarkably consistent across states. Zero-inflation was generally low for most disaster types, indicating few structural zeros, except for hurricanes, where the probability of structural zeros was notably higher.

In Figure 2, fires and tornadoes showed no significant linear trends over the 1989–2019 period (β = −0.03, 95% Confidence Interval (CI) [−0.11, 0.05] and β = −0.03, 95% CI [−0.07, 0.01]; *p* = 0.417 and *p* = 0.257, respectively), while floods, severe storms, hurricanes, and all weather-related disasters combined exhibited significant nonlinear temporal trends, with multiple spline terms reaching statistical significance (*p* < 0.05). The overall shapes of trajectories, derived from the combined contribution of all spline basis functions and their coefficients, revealed complex non-monotonic dynamics. For floods, two negative and highly significant spline terms (β = −2.09, 95% CI [−3.23, −0.95] and β = −1.98, 95% CI [−2.80, −1.16]; *p* = 0.0003 and *p* <0.0001, respectively) indicated an initial decline, followed by a rebound. Severe storms, hurricanes, and total disasters all showed significant nonlinear effects (all *p* < 0.05). Although spline coefficients were positive, differences in their magnitudes led to more complex and non-monotonic trajectories, with disaster counts rising sharply from the early 1990s to the late 2000s, before showing a modest decline after approximately 2005–2010. To present these temporal trends on an interpretable scale, weather-related disasters rose steadily from the early 1990s, increasing from about 0.3–1.2 to roughly 0.5–2.5 around 2010, before tapering to approximately 0.5–1.8 by 2020. Thus, the increase through the 2000s was substantial, followed by a moderate decline. Fire counts generally trended upward, although the magnitude of change varied. For most groups, increases were modest (on the order of 0.05–0.2 over the full period), whereas one trajectory rose more sharply from about 0.3 to over 1.2. Floods showed a distinct pattern, declining from around 0.3–0.8 in 1990 to a low near 0.1–0.2 in the early 2000s, then rebounding to roughly 0.2–1.25 by 2020; in many cases, the later increase offset the earlier decline. Severe storms exhibited one of the largest changes, increasing from about 0.05–0.3 to approximately 0.2–1.3 by the late 2000s, followed by a decline to roughly 0.2–1.0 by 2020. Tornado counts declined over time, from about 0.03–0.20 in 1990 to approximately 0.02–0.04 by 2020, with no evident rebound. Hurricanes showed a similar pattern to severe storms, increasing from 0–0.15 to around 0.1–1.6 by about 2010, then declining to roughly 0.1–0.9 by 2020.

### 3.3. Geographic Clustering of US States Based on Temporal Disaster Trends

**Figure 3:**
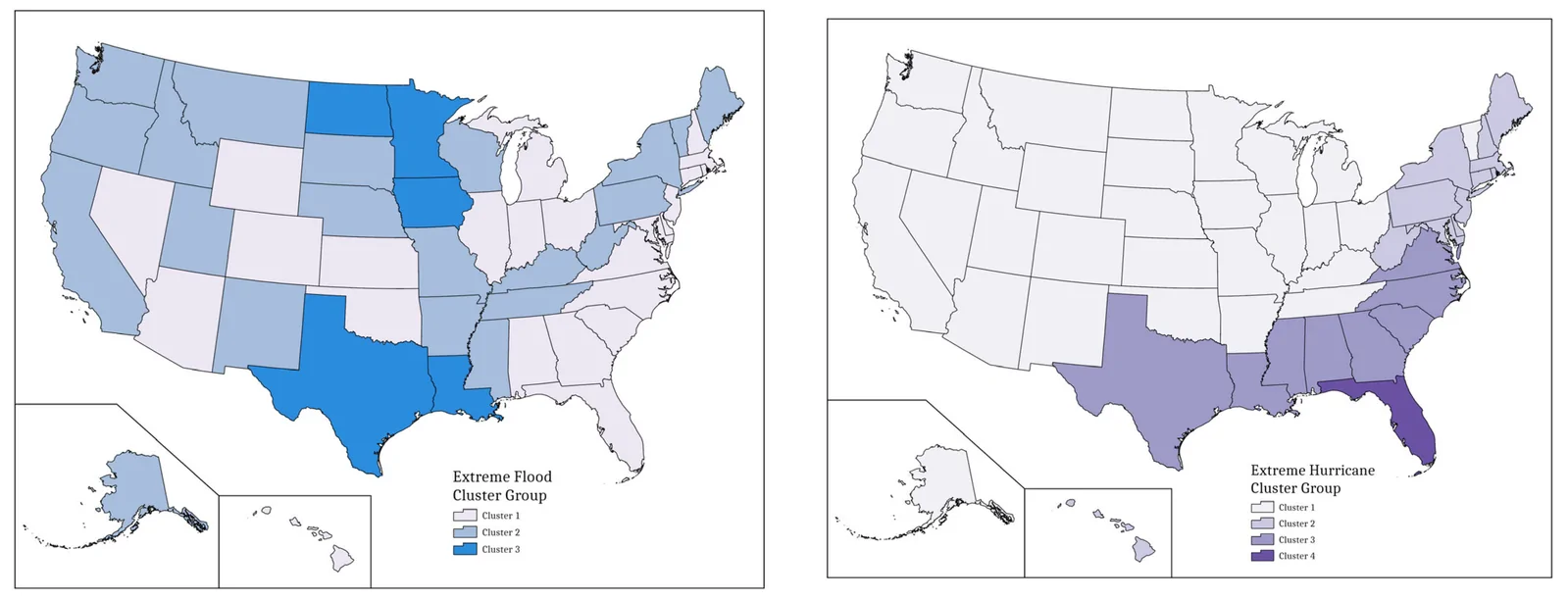

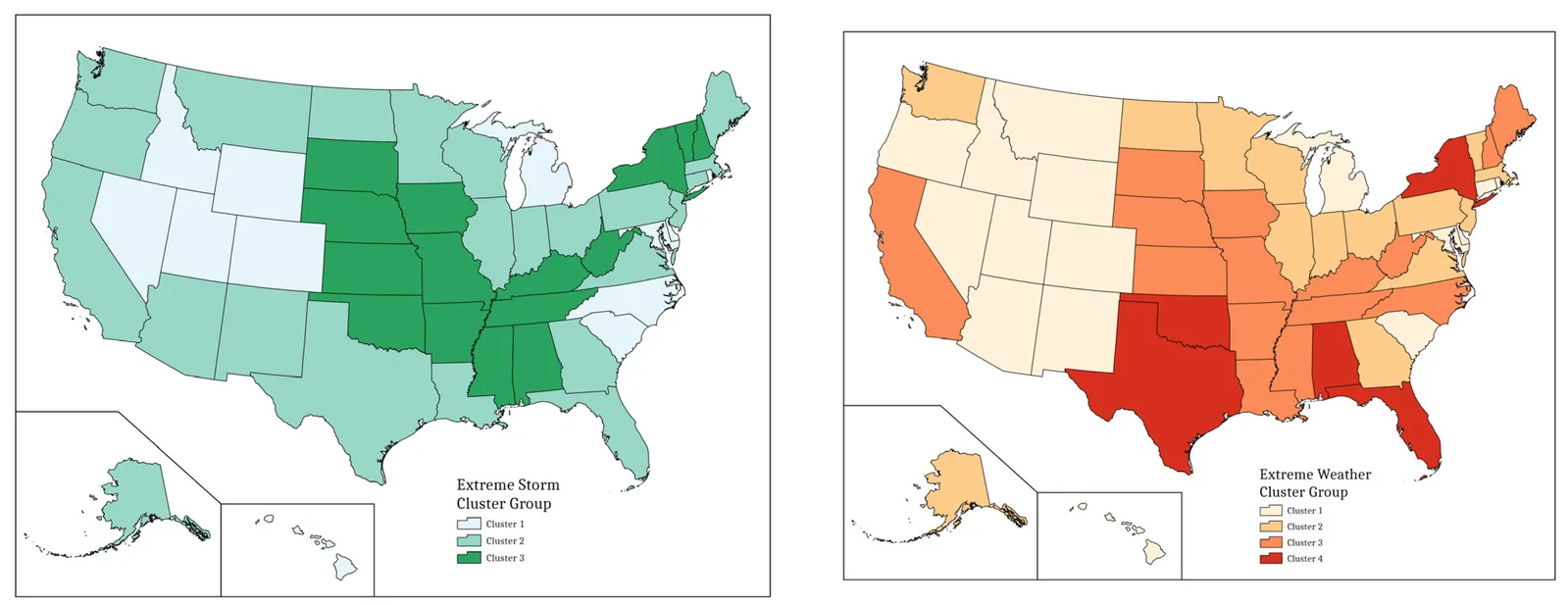
Geographic distribution of disaster occurrences.

Hierarchical clustering was applied to the model predicted disaster occurrence trajectories for floods, severe storms, hurricanes, and all weather-related disasters combined. Predictions were obtained from the final ZIP or ZINB models described above. The optimal number of clusters was determined by visually inspecting the dendrograms and selecting the cut point that balanced between-cluster separation and within-cluster similarity (Figure S3, Supplementary Information). Distinct temporal patterns of the four disaster types are illustrated in Figures S4–S7 (Supplementary Information), and their geographic distributions are mapped in Figure 3.

## 4. Discussion

This study characterized temporal, hazard-specific, and geographic patterns in FEMA emergency and major disaster declarations for weather-related hazards across U.S. states from 1989 to 2019. The observed patterns provide a macro-level baseline that may inform future preparedness research and planning. Rather than a simple monotonic increase, declaration patterns were nonlinear, with substantial interannual variability and pronounced peaks during the mid-2000s and early 2010s, followed by declines that generally remained above early-period levels. Because FEMA declarations reflect events that warranted federal assistance, these findings should be interpreted as patterns in declared disasters, rather than direct measures of hazard frequency, severity, healthcare system readiness, or health impacts.

### 4.1. Interpreting National and Hazard-Specific Trends

Over the study period, overall weather-related declarations and severe storms demonstrated statistically significant nonlinear temporal trajectories trends in annual state-level counts, while tornado-related declarations declined in the national annual analyses. When examined in five-year intervals and with mixed-effects models allowing for nonlinearity, several hazard categories, particularly floods, severe storms, hurricanes, and total weather-related disasters, showed clear nonlinear trajectories. The observed patterns may be useful for anticipating changes in the types of declared weather-related disasters for which regional systems should prepare. However, FEMA declaration data do not measure the clinical, operational, or health consequences of individual events. In addition, this analysis focused on FEMA-declared, generally discrete events and did not evaluate slow-onset hazards such as prolonged heat, drought, or chronic wildfire-smoke exposure. Future research should examine how both discrete and slow-onset climate-related hazards affect healthcare delivery, continuity of care, and population health.

### 4.2. Geographic Heterogeneity and Implications for Preparedness

State-level modeling identified substantial variation in baseline disaster declaration counts and clustering grouped states with similar temporal modeled trajectories. These clusters offer a potential blueprint for regional academic–public health–healthcare partnerships: states with shared hazard profiles could collaborate to identify common preparedness priorities, develop regionally relevant educational resources, coordinate mutual-aid strategies, and plan for continuity of care. This approach does not imply that clustered states have identical risks or capacities; rather, it provides a data-informed starting point for regional assessment and for linking future analyses of hazard patterns with healthcare capacity, workforce readiness, and community vulnerability. Regional partners may use these state-level patterns as an initial planning input, alongside local assessments of healthcare capacity, community vulnerability, and infrastructure needs.

### 4.3. Health Equity: Disaster Trends as a Multiplier of Vulnerability

This study did not include measures of social vulnerability, demographic characteristics, healthcare access, or health outcomes; therefore, it cannot determine whether the identified declaration patterns were associated with inequitable exposure, morbidity, healthcare disruption, or recovery. Nevertheless, prior research has shown that disaster-related health consequences may be unevenly distributed, particularly among communities facing limited access to healthcare, transportation barriers, chronic disease burden, economic constraints, or infrastructure challenges [2,3].

The state-level clusters identified here provide a framework for future linkage analyses. For example, future studies could examine whether states with similar declaration trajectories also share patterns of community vulnerability, healthcare capacity, or disaster-related health outcomes. Such analyses could help determine whether and how regional hazard patterns may inform equity-focused preparedness, resource allocation, and continuity-of-care strategies.

### 4.4. Implications for Nursing Education and the Public Health Workforce

Although this study did not assess nursing competencies, educational programs, or workforce readiness, the identified state-level declaration trajectories and clusters may help inform regionally tailored preparedness education. Academic nursing programs, public health agencies, and healthcare systems serving states with similar hazard profiles could use these findings as a starting point to identify shared priorities for disaster preparedness, continuity of care, risk communication, and interprofessional coordination.

A future data-translation application could link declaration trends and state-level clusters with validated measures of community vulnerability, healthcare capacity, and workforce readiness. Such an approach could support regionally relevant training and preparedness planning; however, its development and effectiveness should be evaluated in future research.

Integrating longitudinal hazard data with metrics of population vulnerability is a critical next step. This approach allows for the identification of regional education gaps and clinical preparedness needs that align with the specific disaster risks faced by healthcare workers in those areas.

### 4.5. Limitations and Future Directions

Several limitations should be considered when interpreting these findings. First, FEMA makes disaster declarations by state or county rather than by unique physical events; consequently, a single storm affecting multiple states results in separate state-level or county-level records. In this analysis, when a full state received a declaration, the data were adjusted to each constituent county to ensure all state events were counted using a consistent geographic weighting. Second, the study did not standardize counts by state population, land area, or density, and therefore should not be interpreted as a direct measure of per capita risk. Third, FEMA declarations primarily capture discrete events that warrant federal assistance and exclude many slow-onset hazards, such as prolonged heat or drought, that also significantly impact public health. Finally, these data do not directly measure hazard severity, health outcomes, or social vulnerability.

Future work should integrate these FEMA declaration trends with validated measures of hospital utilization, mortality, and community-level social vulnerability. Linking regional hazard trajectories with health system capacity and nursing workforce readiness across the identified state clusters remains a critical next step. Evaluating how regionally tailored educational interventions translate into measurable improvements in response performance would further advance the evidence base for climate-informed healthcare preparedness.

## 5. Conclusions

Analysis of FEMA weather-related disaster declarations from 1989–2019 demonstrates that the U.S. declaration patterns varied substantially over time, by hazard type, and across states. Overall declarations, severe storms, floods, and hurricanes showed significant nonlinear trajectories, with pronounced peaks during the mid-2000s and early 2010s, followed by declines that generally remained above early-period levels. Fires and tornadoes did not show significant linear trends. State-level models also identified considerable variation in baseline declaration counts, while hierarchical clustering grouped states with similar modeled temporal trajectories. These findings provide a national, macro-level epidemiologic baseline for describing weather-related disaster declaration patterns under the modern FEMA declaration framework. Because FEMA declarations reflect events that exceeded state, territorial, or tribal response capacity and warranted federal assistance, they should not be interpreted as direct measures of hazard exposure, disaster severity, health outcomes, population vulnerability, or healthcare system performance. Nevertheless, documented temporal and geographic variation offers a practical data framework for preparedness planning. In particular, state-level trajectory clusters can provide a blueprint for regional academic–public health–healthcare partnerships to align training, mutual-aid planning, continuity-of-care strategies, and workforce development with shared and evolving hazard profiles. Future studies should link these declaration patterns with county- and community-level measures of social vulnerability, healthcare capacity, nursing workforce readiness, and health outcomes. Such work can support more equitable, climate-informed preparedness and help nursing and public health systems move from reactive response toward anticipatory, regionally tailored planning.

## Figures and Tables

**Table 1:** Fixed time effects and state-level variability in disaster occurrences, 1989–2019.

Disaster	Model	Fixed Effects					Random Effects	
		Count Part			Zero-Inflation Part		Random Intercept Variance	Random Slope Variance
		Year	β (95% CI)	*p*	Intercept β (95% CI)	*p*	Variance	Variance
Fire	ZIP, linear	Year	−0.03 (−0.11, 0.05)	0.417	−0.02 (−1.22, 1.18)	0.9790	1.7	1.13 × 10^−3^
Flood	ZIP, nonlinear	Spline1	−2.09 (−3.23, −0.95)	0.0003	−0.67 (−1.34, 0)	0.0505	0.14	6.86 × 10^−5^
		Spline2	−1.98 (−2.80, −1.16)	<0.0001				
		Spline3	0.04 (−0.49, 0.57)	0.8875				
Severe storm	ZIP, nonlinear	Spline1	2.44 (1.42, 3.46)	<0.0001	−3.58 (−6.62, −0.54)	0.0204	0.56	1.17 × 10^−3^
		Spline2	2.99 (2.42, 3.56)	<0.0001				
		Spline3	1.86 (1.19, 2.53)	<0.0001				
Tornado	ZIP, linear	Year	−0.03 (−0.07, 0.01)	0.2571	0.58 (−0.67, 1.83)	0.3620	1.23	3.33 × 10^−4^
Hurricane	ZINB, nonlinear	Spline1	2.35 (0.23, 4.47)	0.0302	0.43 (0.12, 0.74)	0.0091	1.42	1.60 × 10^−5^
		Spline2	2.88 (1.80, 3.97)	<0.0001				
		Spline3	1.72 (0.33, 3.11)	0.0157				
All weather-related disasters combined	ZIP, nonlinear	Spline1	0.79 (0.22, 1.36)	0.0068	−2.86 (−3.64, −2.08)	<0.0001	0.30	1.11 × 10^−4^
		Spline2	1.33 (1.00, 1.66)	<0.0001				
		Spline3	0.64 (0.33, 0.95)	0.0001				

## Data Availability

All data used for this work was from U.S. government sources and is already publicly available OpenFEMA Data Sets (https://www.fema.gov/about/openfema/data-sets) (accessed on 12 May 2026).

## Supplementary Materials

The following supporting information can be downloaded at: https://www.mdpi.com/article/10.3390/ijerph23080984/s1, Table S1: Dispersion parameters for different disasters; Table S2: LRT and information criteria of GLMMs with and without spline terms; Figure S1: US States (%) experiencing weather-related disasters by annual count and type (1989–2019); Figure S2: Statewide disaster counts by type, in 5-year intervals from 1990 to 2019; Figure S3: Hierarchical clustering dendrograms of U.S. states based on temporal trends in predicted disaster occurrences; Figure S4: Clusters of US states by temporal trends in predicted all weather-related disaster occurrences; Figure S5: Clusters of US states by temporal trends in predicted flood occurrences; Figure S6: Clusters of US states by temporal trends in predicted severe storm occurrences; Figure S7: Clusters of US states by temporal trends in predicted hurricane occurrences.

## References

[1] NOAA (2023). National Centers for Environmental Information U.S. Billion-Dollar Weather and Climate Disasters. https://www.climate.gov/news-features/blogs/beyond-data/2023-historic-year-us-billion-dollar-weather-and-climate-disasters.

[2] Salas R.N., Burke L.G., Phelan J., Wellenius G.A., Orav E.J., Jha A.K. (2024). Impact of Extreme Weather Events on Healthcare Utilization and Mortality in the United States. Nat. Med..

[3] Dewi S.P., Kasim R., Sutarsa I.N., Dykgraaf S.H. (2024). A Scoping Review of the Impact of Extreme Weather Events on Health Outcomes and Healthcare Utilization in Rural and Remote Areas. BMC Health Serv. Res..

[4] National Academies of Sciences, Engineering, and Medicine (2021). The Future of Nursing 2020–2030: Charting a Path to Achieve Health Equity. https://www.nationalacademies.org/projects/HMD-HCS-18-11/publication/25982.

[5] American Association of Colleges of Nursing 2026 Edition The Essentials: Core Competencies for Professional Nursing Education. https://www.aacnnursing.org/Portals/0/PDFs/Publications/Essentials-2026.pdf.

[6] Joint Commission National Performance Goal #3: Emergency Readiness|Joint Commission. https://www.jointcommission.org/en-us/standards/national-performance-goals/emergency-readiness.

[7] American Nurses Association Who Will Be There? Ethics, the Law, and a Nurse’s Duty to Respond in a Disaster. https://www.nursingworld.org/globalassets/docs/ana/who-will-be-there_disaster-preparedness_2017.pdf.

[8] Khorram-Manesh A., Mani Z. (2025). Navigating the Chaos: A Scoping Review of Gaps in Disaster Nursing and a Roadmap for The Future. BMC Nurs..

[9] Federal Emergency Management Agency Disaster Declaration Process. https://www.fema.gov/pdf/media/factsheets/dad_disaster_declaration.pdf.

[10] Federal Emergency Management Agency (2022). IPAWS Strategic Plan FY 2022–2026.

[11] Stalhandske Z., de Ruiter M.C., Chambers J., Zimmermann S., Colón-González F.J., Sairam N., Bresch D.N., Kropf C.M. (2025). Global Assessment of Population Exposure to Multiple Climate-Related Hazards from 2003 to 2021: A Retrospective Analysis. Lancet Planet. Health.

[12] Leppold C., Gibbs L., Block K., Reifels L., Quinn P. (2022). Public Health Implications of Multiple Disaster Exposures. Lancet Public Health.

[13] Kruger K., Brysiewicz P., Lori J., Bell S.A. (2025). The Role of the Nursing Workforce in Health System Resilience during Disasters: A Scoping Review of Empirical Studies. Int. J. Nurs. Stud. Adv..

[14] Hassan S., Wiciak M., Escobar K., Montalvo M.d.M.G., Richards T., Villanueva H., Ortiz J., Evans D.P., Nunez-Smith M. (2024). Understanding Barriers and Facilitators to Disaster Preparedness in Federally Qualified Health Centers in the United States: A Mixed Methods Study. Disaster Med. Public Health Prep..

[15] Ginex P., Dickman E., Elia M.R., Burbage D., Wilson R., Koos J.A., Sivakumaran K., Morgan R.L. (2023). Climate Disasters and Oncology Care: A Systematic Review of Effects on Patients, Healthcare Professionals, and Health Systems. Support. Care Cancer.

[16] Horn D.P., Lee E.A., Lindsay B.R., Painter W.L., Reese S., Stienstra L.R., Webster E.M. (2022). FEMA’s Role in the COVID-19 Federal Pandemic Response.

[17] 17. U.S. Department of Homeland Security, Office of the Inspector General (2021). Lessons Learned from FEMA’s Initial Response to COVID-19. https://www.oig.dhs.gov/sites/default/files/assets/2021-09/OIG-21-64-Sep21.pdf.

[18] Ward J.H. (1963). Hierarchical Grouping to Optimize an Objective Function. J. Am. Stat. Assoc..

[19] Strauss T., von Maltitz M.J. (2017). Generalising Ward’s Method for Use with Manhattan Distances. PLoS ONE.

