# Supplementary material for: Temporal Trends and Geographic Clustering of U.S. Weather-Related Disasters (1989–2019): A Foundational Baseline for Healthcare Preparedness
Source: Int J Environ Res Public Health. 2026 Jul 29;23(8):984. doi: 10.3390/ijerph23080984 (PMC13512037; doi:10.3390/ijerph23080984)
Supplement: Supplementary file 1 [file ijerph-23-00984-s001.zip › Figure S2.pdf]

Figure S2. Statewide disaster counts by type, in 5-year intervals from 1990 to 2019

Note: Each bar represents the number of statewide disaster occurrences within a 5-year period from 1990 to 2019. Data from 1989 were excluded in this analysis for consistent time interval. Counts reflect one record per disaster per state across the US. The six panels display counts for all weather-related disasters combined and for individual categories: fires, floods, severe storms, tornadoes, and hurricanes.

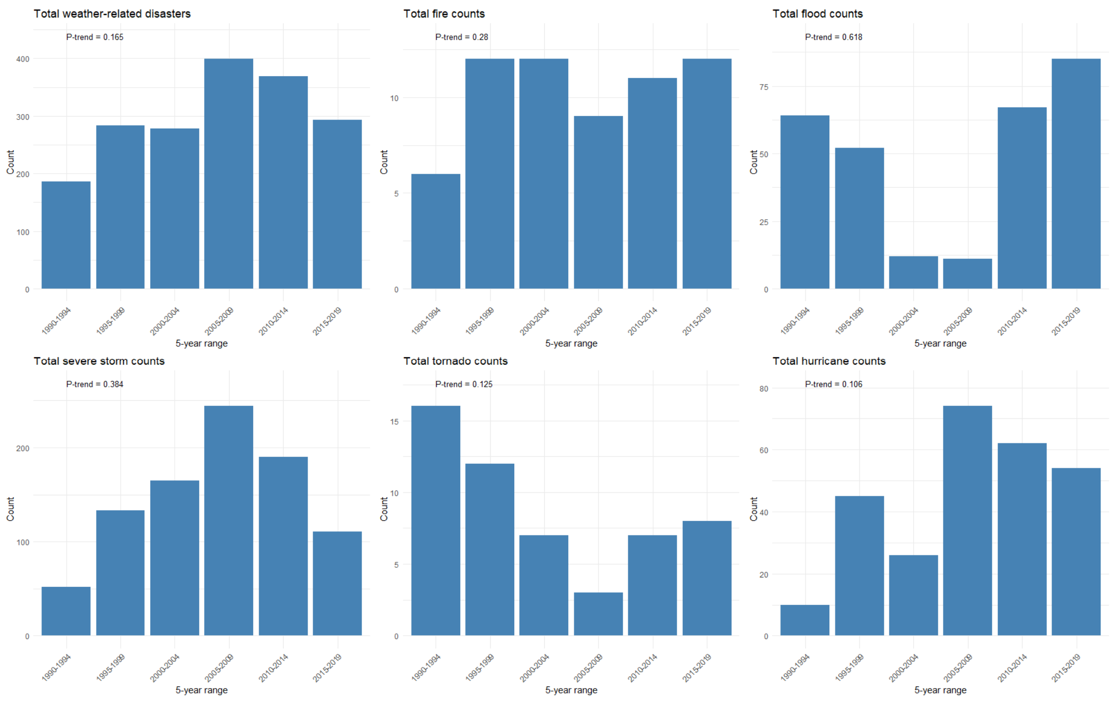

Note: Each disaster count is counted once across the US. Data from 1989 is not included in this figure.
