# Supplementary material for: Temporal Trends and Geographic Clustering of U.S. Weather-Related Disasters (1989–2019): A Foundational Baseline for Healthcare Preparedness
Source: Int J Environ Res Public Health. 2026 Jul 29;23(8):984. doi: 10.3390/ijerph23080984 (PMC13512037; doi:10.3390/ijerph23080984)

Figure S3. Hierarchical clustering dendrograms of U.S. states based on temporal trends in predicted disaster occurrences.

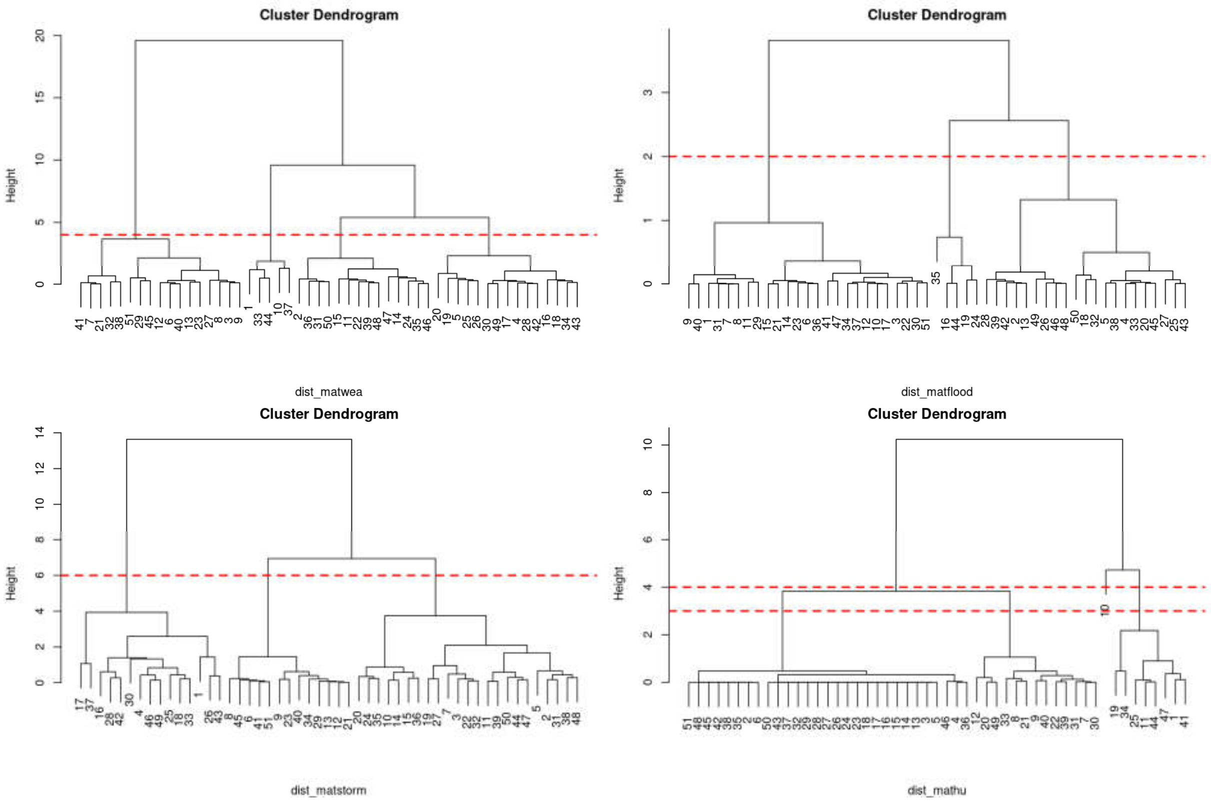

Supplement: Supplementary file 1 [file ijerph-23-00984-s001.zip › Figure S3.pdf]
