# Supplementary material for: Temporal Trends and Geographic Clustering of U.S. Weather-Related Disasters (1989–2019): A Foundational Baseline for Healthcare Preparedness
Source: Int J Environ Res Public Health. 2026 Jul 29;23(8):984. doi: 10.3390/ijerph23080984 (PMC13512037; doi:10.3390/ijerph23080984)
Supplement: Supplementary file 1 [file ijerph-23-00984-s001.zip › Table S1.pdf]

Table S1. Dispersion parameters for different disasters.

Disaster type	Dispersion parameter
Fire	1.47
Flood	1.31
Severe storm	1.32
Tornado	1.15
Hurricane	1.85
All weather-related disasters combined	1.38
