# Supplementary material for: Temporal Trends and Geographic Clustering of U.S. Weather-Related Disasters (1989–2019): A Foundational Baseline for Healthcare Preparedness
Source: Int J Environ Res Public Health. 2026 Jul 29;23(8):984. doi: 10.3390/ijerph23080984 (PMC13512037; doi:10.3390/ijerph23080984)
Supplement: Supplementary file 1 [file ijerph-23-00984-s001.zip › Table S2.pdf]

Table S2. LRT and information criteria of GLMMs with and without spline terms.

	DF	AIC	BIC	LRT_vs_Linear model	
				Chi-square	P
Fire					
**Linear**	**6**	**433.95**	**466.14**		
Linear + spline	8	434.17	477.09	3.78	0.1513
Spline only	8	NA	NA		
Flood					
Linear	6	1666.56	1698.75		
Linear + spline	8	NA	NA	NA	NA
**Spline only**	**8**	**1602.15**	**1745.07**	**68.41**	**<0.0001**
Severe storm					
Linear	6	3121.7	3153.9		
Linear + spline	8	2968.73	3011.66	156.97	<0.0001
**Spline only**	**8**	**2968.73**	**3011.66**	**156.97**	**<0.0001**
Tornado					
Linear	6	493.43	525.63		
Linear + spline	8	NA	NA	NA	NA
Spline only	8	492.51	535.43	4.92	0.0853
Hurricane					
Linear	7	NA	NA		
Linear + spline	9	1285.01	1333.31	NA	NA
Spline only	9	1285.01	1333.3	NA	NA
All weather-related disasters combined					
Linear	6	2184.79	2222.35		
Linear + spline	8	4447.6	4490.5	50.09	<0.0001
**Spline only**	**8**	**4447.6**	**4490.5**	**50.09**	**<0.0001**

Abbreviations: LRT, likelihood ratio test; GLMM, generalized linear mixed model; DF, degree of freedom; AIC, Akaike Information Criterion; BIC, Bayesian Information Criterion.
Note: The “Linear” model includes year as a linear term; the “Linear + spline” model includes both the linear term and basic spline terms (df = 3) for year; the “Spline only” model includes only spline terms for year. LRT_vs_Linear compares the fit of the linear model with the other two models with spline terms. P values from LRT tests <0.05 indicate a statistically significant improvement of fit. Lower AIC and BIC values indicate better model fit. Bold values indicate the preferred model for each disaster type. “NA” indicates AIC/BIC values not returned due to model convergence problem.
